# Identification of Glycine Receptor α3 as a Colchicine-Binding Protein

**DOI:** 10.3389/fphar.2018.01238

**Published:** 2018-11-08

**Authors:** Xikun Zhou, Mingbo Wu, Yongmei Xie, Guo-Bo Li, Tao Li, Rou Xie, Kailun Wang, Yige Zhang, Chaoyu Zou, Wenling Wu, Qi Wang, Xiangwei Wang, Ximu Zhang, Jiong Li, Jing Li, Yu-Quan Wei

**Affiliations:** ^1^State Key Laboratory of Biotherapy and Cancer Center, West China Hospital, Sichuan University and Collaborative Innovation Center of Biotherapy, Chengdu, China; ^2^School of Bioscience and Biotechnology, Chengdu Medical College, Chengdu, China; ^3^Key Laboratory of Obstetrics and Gynecologic and Pediatric Diseases and Birth Defects of Ministry of Education, Department of Gynecology and Obstetrics, West China Second University Hospital, Sichuan University, Chengdu, China; ^4^State Key Laboratory of Oral Diseases, National Clinical Research Center for Oral Diseases, West China Hospital of Stomatology, Sichuan University, Chengdu, China

**Keywords:** colchicine, glycine receptor alpha 3, inflammatory pain, gouty arthritis, virtual target identification

## Abstract

Colchicine (Col) is considered a kind of highly effective alkaloid for preventing and treating acute gout attacks (flares). However, little is known about the underlying mechanism of Col in pain treatment. We have previously developed a customized virtual target identification method, termed IFPTarget, for small-molecule target identification. In this study, by using IFPTarget and ligand similarity ensemble approach (SEA), we show that the glycine receptor alpha 3 (GlyRα3), which play a key role in the processing of inflammatory pain, is a potential target of Col. Moreover, Col binds directly to the GlyRα3 as determined by the immunoprecipitation and bio-layer interferometry assays using the synthesized Col-biotin conjugate (linked Col and biotin with polyethylene glycol). These results suggest that GlyRα3 may mediate Col-induced suppression of inflammatory pain. However, whether GlyRα3 is the functional target of Col and serves as potential therapeutic target in gouty arthritis requires further investigations.

## Introduction

Gouty arthritis is a type of inflammatory disease caused by the deposition of monosodium urate (MSU) crystals (mainly within joints), and is associated with numerous comorbidities, such as kidney disease, hypertension, obesity, diabetes, and cardiovascular diseases, that negatively impact the long-term prognosis and quality of life (Drug Therapeutics Bulletin, 2018). This kind of inflammation will be initiated when MSU interacts with resident macrophages and consequently recruits more neutrophils to the inflammatory sites. The activation of NLRP3 inflammasome and subsequent release of interleukin 1β are key pathophysiologic features of acute gouty arthritis (Dalbeth et al., 2016). The main symptoms of gout flares are severe inflammatory pain, swelling, heat, redness of the affected joint, and restriction of joint movement (Dalbeth et al., 2016). Eliminating gout flares and preventing joint damage are considered two major focuses of therapeutic intervention in gout.

Non-steroidal anti-inflammatory drugs and colchicine (Col) remain the most widely recommended drugs for the treatment of acute flares (Dalbeth et al., 2016; Richette et al., 2017). Col is a tricyclic and lipid-soluble alkaloid, and also is one of the oldest drugs still available for acute flares of gout, familial Mediterranean fever, and a variety of rheumatologic and cardiovascular diseases (Slobodnick et al., 2015). Recent studies have suggested that Col can disrupt microtubule polymerization, microtubule-derived spatial arrangement of mitochondria, and suppress the migration of neutrophils associated with mediating some gout symptoms (Misawa et al., 2013). However, the mechanism by which Col prevents MSU crystal-induced inflammation pain is still less well-understood. Therefore, identifying the other binding targets of Col will further elucidate the mechanisms of Col's anti-inflammatory properties.

Number of computational methods have been currently developed. A similarity ensemble approach (SEA) based on quantitating the similarity of 2D fingerprints describing the structures of their ligands successfully identified new drug-target associations (Keiser et al., 2009). We previously established a new customized virtual target identification method termed IFPTarget, in which a protein-ligand interaction fingerprinting (IFP) method was used to analyze the target-specific binding features (Li et al., 2013, 2017). In this study, we used IFPTarget and SEA to predict the potential targets for Col and have demonstrated that Col binds directly to the glycine receptor alpha 3 (GlyRα3).

## Materials and methods

### Cells

THP-1 and 293T cells were gotten from American Type Culture Collection (Manassas, VA) and cultured in RPMI 1640 and DMEM medium (HyClone, GE Healthcare Life Sciences, USA) supplemented with 10% heat-inactivated FBS (ST30-3302, PAN, Germany) and penicillin (100 U/ml)-streptomycin (100 μg/ml) (HyClone), respectively. The cell line has been tested without of mycoplasma contamination.

### Target prediction by IFPTarget

A virtual target screening system, termed IFPTarget (Li et al., 2017), was employed to predict the potential binding targets of Col. Using IFPTarget, Col was screened against the target database, containing >11,900 protein structures covering >2,800 protein targets. The possible target hits for Col were ranked by a comprehensive index C-value (Li et al., 2013, 2017). The top 1% ranked target “hits” identified by IFPTarget are given in Table S1.

### Statistical analysis

Statistical analysis and plotting graphs performed by GraphPad Prism 6.0 software. Statistical analysis was evaluated by 2-tailed Student's *t*-test for comparing two groups. Data are presented as mean ± SDEVs and significance was designated as *p* < 0.05.

## Results

### GlyRα3 is the potential target of col

**Figure 1:**
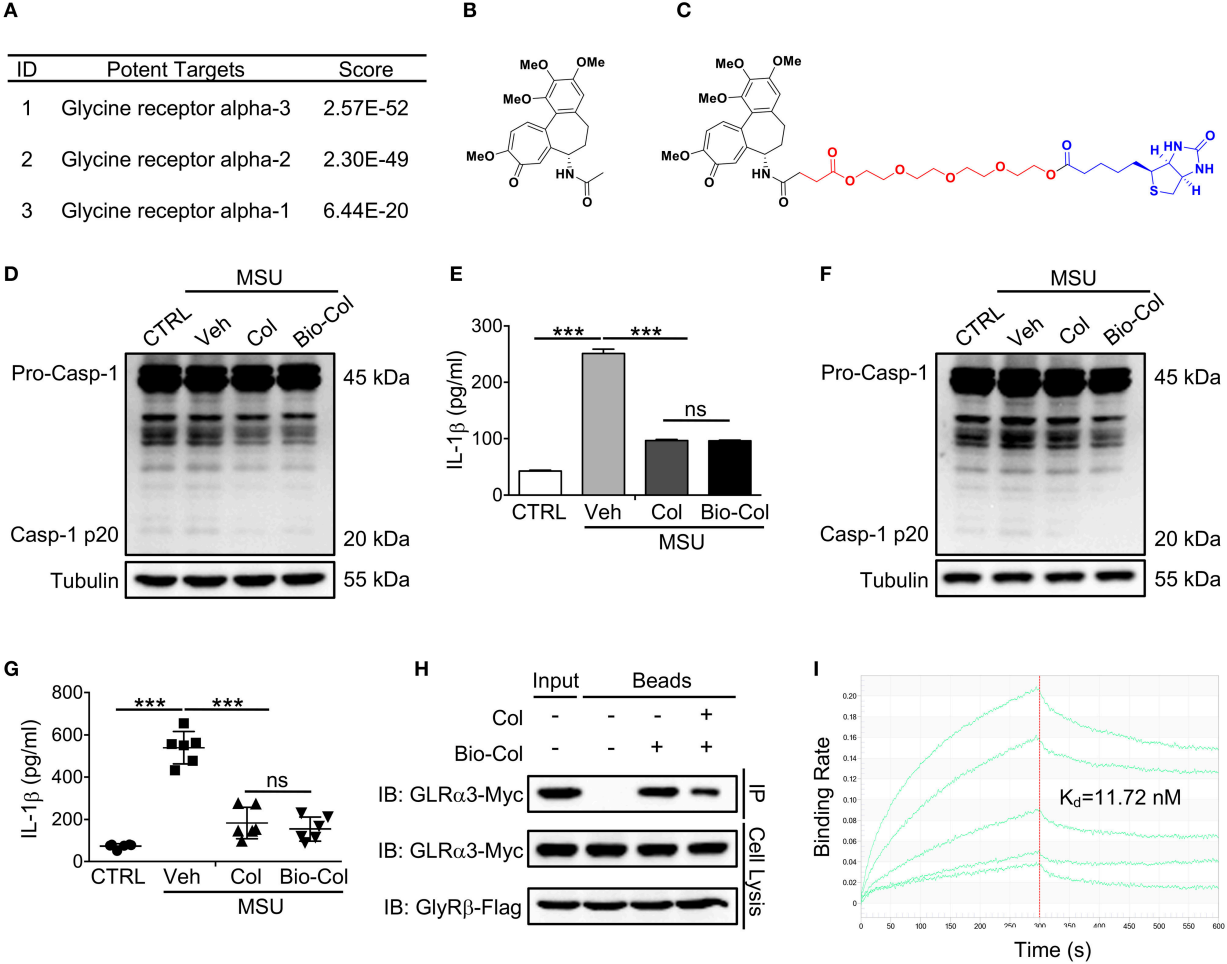
GlyRα3 is a binding protein of Col. **(A)** Part of the predicted targets for Col. **(B,C)** Molecular structure of Col **(B)** and Bio-Col **(C)**. **(D,E)** Effect of caspase-1 activation **(D)** and IL-1β production **(E)** of the Col and Bio-Col in MSU-treated THP-1 cells. ELISA analysis of IL-1β protein levels in the cell culture medium 6 h after MSU treatment. **(F,G)** Effect of caspase-1 activation **(F)** and IL-1β production **(G)** of the Col and Bio-Col in MSU-treated mice. The air pouch model was established and 2 mg of MSU crystals was injected into the air pouches. After 6 h, the air pouch fluid was harvested by injecting 3 ml of PBS. The cell pellet was used for the detection of cleaved caspase-1 p20. The supernatants of the air pouch fluid were analyzed by ELISA for IL-1β. **(H)** GlyRα3 was affinity-purified from the cell lysis of GlyRα3-Myc and GlyRβ-Flag overexpressed 293T cells using Bio-Col immobilized beads. The SDS-PAGE gel was immunoblotted with anti-Myc antibody. **(I)** Biolayer interferometry analysis of the binding of recombinant GlyRα3 to Bio-Col. The streptavidin biosensor tips of the FortéBio were coated with Bio-Col. Applied analyte concentrations were range between 0.1 and 1,000 nM. The sensorgrams are shown in green lines. K_d_ values were calculated from steady-state values. Veh, Vehicle. These data are representative of three experiments or six mice per group and are shown as the means ± SDEVs (ns, not significant. ^***^p < 0.001 by the *t*-test).

Our IFPTarget program uses an interaction fingerprinting method for target-specific interaction analyses and a comprehensive index C-value for target ranking (Li et al., 2013). Here, we used IFPTarget to predict the potential targets for Col against an in-house target database, which contains more than 11,900 protein structures covering >2,800 protein targets (Li et al., 2017), and the predicted targets were ranked by Cvalue. The top 1% target “hits” identified by IFPTarget are given in Table S1. In addition, we used the SEA method to predict the targets for Col (Keiser et al., 2009). Consistent with previously reported (Misawa et al., 2013), tubulin proteins were ranked at the top of the list (Figure 1A and Tables S1, S2). Interestingly, glycine receptor alpha 3 (GlyRα3) had the highest predicted score with Col except tubulins, followed by GlyRα2 and GlyRα1. GlyRα3 is a member of the cysteine loop (Cys-loop) superfamily of ligand-gated ion channels and has been identified to have broader implications in inflammatory pain modulation (Harvey et al., 2004; Xiong et al., 2012). We hypothesized that Col-attenuated MSU crystal-induced inflammatory pain may work through binding to GlyRα3.

### Synthesis of col-biotin conjugate

To evaluate the potential binding of Col to GlyRα3, we synthesized a Col-biotin conjugate (Bio-Col) by linking Col and biotin with polyethylene glycol (PEG) (Figures 1B,C and Figure S1). The structure was validated by NMR spectrometer (Figures S2, S3). As the action mechanism of Col in the treatment of gout is mainly involves inflammatory processes (e.g., inflammasome and IL-1β), the effect of Bio-Col on caspase-1 activation and IL-1β production were further investigated to verify if this conjugate has the same biological activity as unlabeled Col. In an *in vitro* model of the PMA-inducible THP-1 cells, Bio-Col was found to inhibit caspase-1 activation (p20 level) and IL-1β production same as Col after the treatment of 150 μg/ml MSU (Figures 1D,E). The subcutaneous air-pouch model is a widely-used animal model for MSU-induced acute gouty inflammation (Torres et al., 2009; Hoffman et al., 2010; Uratsuji et al., 2012). A mouse air pouch model was established to validate the biological activity of Bio-Col in inflammation in response to MSU crystals *in vivo*. The backs of mice were subcutaneously injected with sterile air twice in 1 week, and 2 mg of MSU crystals in 0.5 ml of PBS or 0.5 ml of PBS alone was injected into the air pouches. The air pouch fluids were lavaged by 3 ml of PBS. Notably, both the expression levels of caspase-1 p20 and IL-1β in Col or Bio-Col treated mice were significantly decreased compared to that of MSU group (Figures 1F,G). These data suggested that Bio-Col and unlabeled Col had an inhibitory role on inflammasome activation, and could be used to identify the binding of GlyRα3 to Col.

### GlyRα3 directly binds to col

It is reported that the native GlyRs are pentameric receptors composed of 2α and 3β subunits (Betz and Laube, 2006). Bio-Col was first conjugated to streptavidin beads, and the immobilized Bio-Col was then incubated with the cell lysis of GlyRα3 and GlyRβ over-expressed 293T cells. The Bio-Col-binding proteins were pulled down by streptavidin beads and separated by denaturing gel electrophoresis. As expected, the results of western blotting showed that GlyRα3 could be affinity-purified with Bio-Col-immobilized beads, and this could be reversed when free Col was added into lysates prior to incubation with streptavidin beads (Figure 1H). We next prepared the purified recombinant GlyRα3 protein using a prokaryotic expression system. The Bio-Col and GlyRα3 protein binding interactions were characterized on streptavidin biosensors by the Octet K2 system. The quantitative binding assays showed that Bio-Col could indeed bind to GlyRα3 with a K_d_ of 4.221 nM (Figure 1I).

## Discussion

Col is a well-known oldest drug and useful in treating many other disorders, and dissection of its mechanism of action has been an area of active investigation. In this study, we demonstrate that Col binds directly to the GlyRα3.

Formerly small-molecule target identification experiments, such as direct biochemical and genetic interaction methods, are relatively costly and time-consuming (Ziegler et al., 2013). The computational approaches were proved to substantially improve the efficiency of target identification (Katsila et al., 2016). IFPTarget we developed previously combines a target-specific interaction fingerprinting method and a comprehensive index-based target ranking method. The property of supporting parallel computing on multi-core processors make it suitable to predict a great number of protein targets (Li et al., 2017). And our results suggest that it can has a higher accuracy when joint with the chemical similarity approach.

Previous studies have shown that GlyRα3 is a molecular target in inflammatory pain therapy (Harvey et al., 2004; Xiong et al., 2012). Our study provides another evidence for the potential application of GlyRα3. However, there are limitations in this study that need to be mentioned. First, our study does not rule out whether GlyRα3 is the functionally target of Col in MSU crystal-induced inflammatory pain. If it can be verified, this interaction is likely to explain, at least in part, why Col is a clinically effective therapy for patients with acute gout flares. The identification of GlyRα3 as a direct target of Col may open new perspectives for optimizing the use of this ancient remedy in future acute gouty arthritis therapies. Furthermore, it also has not been validated that if there are other mechanisms of Col action, particularly with other glycine receptors. The binding activity of Col with GlyRα1 and GlyRα2 and its function in acute flares will be further investigated in a future study. Moreover, whether the proven ligands, such as strychnine and AM-1488, play a regulatory role in inflammatory pain still needs to be investigated. Therefore, further elucidating the mechanisms of Col's anti-inflammatory pain properties may be beneficial in treating or preventing a variety of illnesses, including gouty arthritis and other rheumatic diseases.

In summary, we predicted the potential targets for Col using IFPTarget and SEA methods, and described the direct interaction of Col and GlyRα3 in this study. Functional characterization of GlyRα3 in inflammatory pain model is still in progress and need be validated.

## Ethics statement

The animal studies were approved by the Ethics Committee of the State Key Laboratory of Biotherapy, Sichuan University. All animal experimental procedures including care, treatment, and killing were accordance with the animal care and institutional guidelines.

## Author contributions

XikZ and Y-QW conceived the study. XikZ and JinL. designed studies and wrote the paper. MW, YX, G-BL, TL, RX, KW, YZ, CZ, WW, XimZ, QW, and XW performed experiments. JioL provided critical resources and reagents.

### Conflict of interest statement

The authors declare that the research was conducted in the absence of any commercial or financial relationships that could be construed as a potential conflict of interest.

## Abbreviations

Col: Colchicine
Bio-Col: Col-biotin conjugate
GlyRα3: glycine receptor α3
MSU: monosodium urate
PEG: polyethylene glycol
PMA: Phorbol 12-myristate 13-acetate
IL-1β: Interleukin 1 beta.
