# Supplementary material for: Identification of Glycine Receptor α3 as a Colchicine-Binding Protein
Source: Front Pharmacol. 2018 Nov 8;9:1238. doi: 10.3389/fphar.2018.01238 (PMC6236057; doi:10.3389/fphar.2018.01238)
Supplement: Supplementary file 1 [file Data_Sheet_1.pdf]

Supplementary Material

Identification of glycine receptor α3 as a colchicine-binding protein

Xikun Zhou[1,†,*], Mingbo Wu[2,†], Yongmei Xie[1,†], Guo-Bo Li[1], Tao Li[3], Rou Xie[1], Kailun Wang[1], Yige Zhang[1], Chaoyu Zou[1], Wenling Wu[1], Qi Wang[1], Xiangwei Wang[1], Ximu Zhang[4], Jiong Li[1], Jing Li[4,*], Yu-Quan Wei[1]

[1]State Key Laboratory of Biotherapy and Cancer Center, West China Hospital, Sichuan University and Collaborative Innovation Center of Biotherapy, Chengdu, 610041, China;

[2]School of Bioscience and Biotechnology, Chengdu Medical College, Chengdu 610500, China;

[3]Department of Gynecology and Obstetrics, Key Laboratory of Obstetrics and Gynecologic and Pediatric Diseases and Birth Defects of Ministry of Education, West China Second University Hospital, Sichuan University, Chengdu 610041, China;

[4]State Key Laboratory of Oral Diseases, National Clinical Research Center for Oral Diseases, West China Hospital of Stomatology, Sichuan University, Chengdu 610041, China

[*]These authors contributed equally to this work.

Correspondence: xikunzhou@scu.edu.cn or lijing1984@scu.edu.cn

Running Title: GlyRα3 is a binding protein of colchicine

Supplementary Material and Methods

Mice. 8-week-old male C57BL/6 mice were purchased from Dossy experimental animals company (Chengdu, China). The animal studies were approved by the Ethics Committee of the State Key Laboratory of Biotherapy, Sichuan University. All animal experimental procedures including care, treatment, and killing were accordance with the animal care and institutional guidelines.

Inflammasome activation. THP-1 cells were exposed to 100 ng/ml phorbol myristate acetate (PMA, S1819, Beyotime Biotechnology, Shanghai, China) on the day before the indicated experiment. The cell medium was replaced with RPMI 1640 medium without FBS and primed with 100 ng/ml LPS (L3129, Sigma, USA) for 3 hours. 5 μM Col or Bio-Col were added into the medium for 30 min and then THP-1 cells were treated with 150 μg/ml monosodium urate (MSU) crystals (tlrl-msu, InvivoGen, San Diego, CA, USA) for another 6 hours. The cell supernatant was collected, IL-1β protein level was detected with ELISA (EHC002b.96, Neobioscience, Shenzhen, China) and cleaved caspase-1 p20 (AG-20B-0042-C100, AdipoGen Life Sciences, San Diego, CA, USA) in cell lysis was detected by western blot analysis.

Mice air pouch model. The backs of mice were subcutaneously injected with 2 ml sterile air and followed by a second injection of 3 ml of sterile air after 3 days. Six days after the first injection, 2 mg MSU crystals in 0.5 ml PBS or 0.5 ml PBS alone was injected into the air pouches. After 6 hours, the mice were anesthetized, and the air pouch fluids were lavaged with 3 ml PBS. The lavages were centrifuged at 1000 G for 5 minutes. The supernatants were used for IL-1β detection using ELISA (EMC001b.96, Neobioscience) according manufacturer's introduction. The cell extract was used for the detection of cleaved caspase-1 p20.

Plasmid construction. For eukaryotic expression, human GlyRα3 and GlyRβ cDNA (provided by Dr. Jiahuai Han, Xiamen University, China) was cloned into a pmaxCloning vector (VDC-1040, Lonza, Cologne, Germany) between restriction sites KpnI and XhoI with an Myc tag

(GlyRα3) or Flag tag (GlyRβ). For prokaryotic expression, human GlyRα3 cDNA was cloned into a pGEX-4T-1 vector (GE Healthcare, Pittsburgh, PA, USA) between restriction sites EcoRI and NotI. All the constructs were confirmed by DNA sequencing.

Streptavidin agarose affinity assay. pmax-GlyRα3-Myc and pmax-GlyRβ-Flag were co-expressed in 293T cells. The Streptavidin Mag Sepharose (28-9857-38, GE Healthcare, Pittsburgh, PA, USA) were equilibrated with RIPA buffer (P0013, Beyotime Biotechnology) and incubated with 1mM Bio-Col for 2 hours at room temperature. After washing with RIPA buffer, Bio-Col conjugated streptavidin beads were further incubated with 293T cell lysates overnight at 4 ℃. In competitive experiments, 0.1 mM Col was added to cell extracts before incubation with the beads. After washing with RIPA buffer, streptavidin beads were resuspended in 1× SDS loading buffer. The pull-down products were detected with anti-Myc antibody (390003, Zen Bioscience, Chengdu, China) by western blot analysis.

Synthesis of Col-biotin conjugate. The biotin conjugate was synthesized by coupling of Col (Sigma, St Louis, MO, USA) with the biotin. Bio-PEG-SA, N-ethyl-N′-(3-dimethylaminopropyl)carbodiimide hydrochloride (EDCI) and 4-dimethylaminopyridine (DMAP) were purchased from Chengdu JuHui Chemical Technology Co., Ltd.(Chengdu, China). N,N-Dimethylformamide (DMF) was supplied by Sinopharm Chemical Reagent Co. Ltd. (Shanghai, China). Deacetylcolchicine was prepared according to the procedure described before (Lagnoux et al., 2005). To a solution of Bio-PEG-SA (100mg, 0.192 mmol) in 30 mL DMF, EDCI (130mg, 6.78 mmol) and DMAP (10mg, 0.082 mmol) were added sequentially. After being stirred at room temperature for 30 min, deacetylcolchicine (80mg, 0.22 mmol) was added and the resulting solution was stirred for an additional 12 h. The solvent was evaporated and the residue was purified by column chromatography (silica gel, DCM/MeOH=1:10) to give Bio-Col as a solid (100 mg, 60.7%) (Figure S1). ^{1}HNMR and ^{13}CNMR spectra were measured on a Bruker AV-400 (400 MHz) NMR spectrometer (Figure S2, S3).

Binding affinity measurements by bio-layer interferometry. The binding affinities of recombinant human GlyRα3 protein to Bio-Col were measured by biolayer interferometry on an Octet K2 System (Pall FortéBio, Menlo Park, CA). Prokaryotic pGEX-4T-1 and pGEX-GlyRα3 plasmid was transformed into *E.coli* BL21 (DE3) cells. The bacteria were grown in LB media and protein expression were induced by 0.5 mM IPTG for 4-5 hours. The glutathione S-transferase (GST) control and GST-GlyRα3 fusion proteins were purified by MagneGST™ Glutathione Particles (V8611, Promega, Madison, WI, USA) and gradient eluted by 1-50 mM glutathione (GSH). Bio-Col was loaded onto Streptavidin (SA) biosensors (18-5021, Pall FortéBio). All affinity measurements were carried out in stabilization buffer (1X PBS, 0.002% Tween-20, and 0.01% BSA). Association was analyzed at various concentrations of recombinant human GlyRα3 proteins (1:10 dilutions starting from 1000 nM to 0.1 nM), followed by measuring dissociation in buffer. Binding kinetics were calculated using the FortéBio Data Analysis v9.0 software. Dissociation constant (Kd) values were obtained from steady-state binding analysis.

Supplementary References

Keiser, M.J., Setola, V., Irwin, J.J., Laggner, C., Abbas, A.I., Hufeisen, S.J., Jensen, N.H., Kuijer, M.B., Matos, R.C., Tran, T.B., et al. (2009). Predicting new molecular targets for known drugs. Nature 462, 175-181.

Lagnoux, D., Darbre, T., Schmitz, M.L., and Reymond, J.L. (2005). Inhibition of mitosis by glycopeptide dendrimer conjugates of colchicine. Chemistry 11, 3941-3950.

Supplementary Figures and Figure Legends

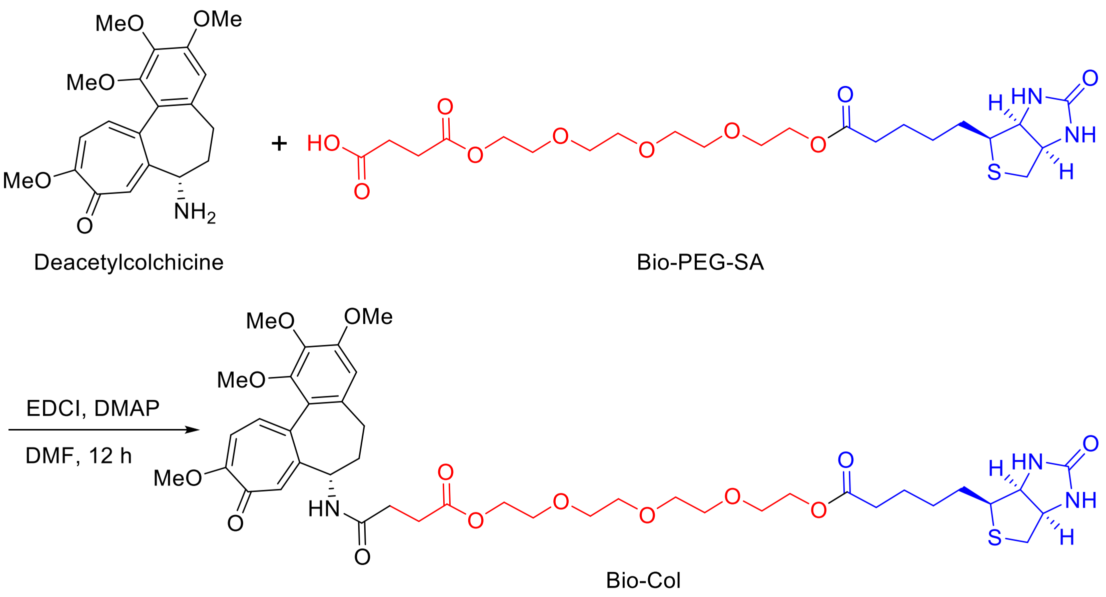

Figure S1. Schematic representation of synthesis of Col-biotin conjugate.

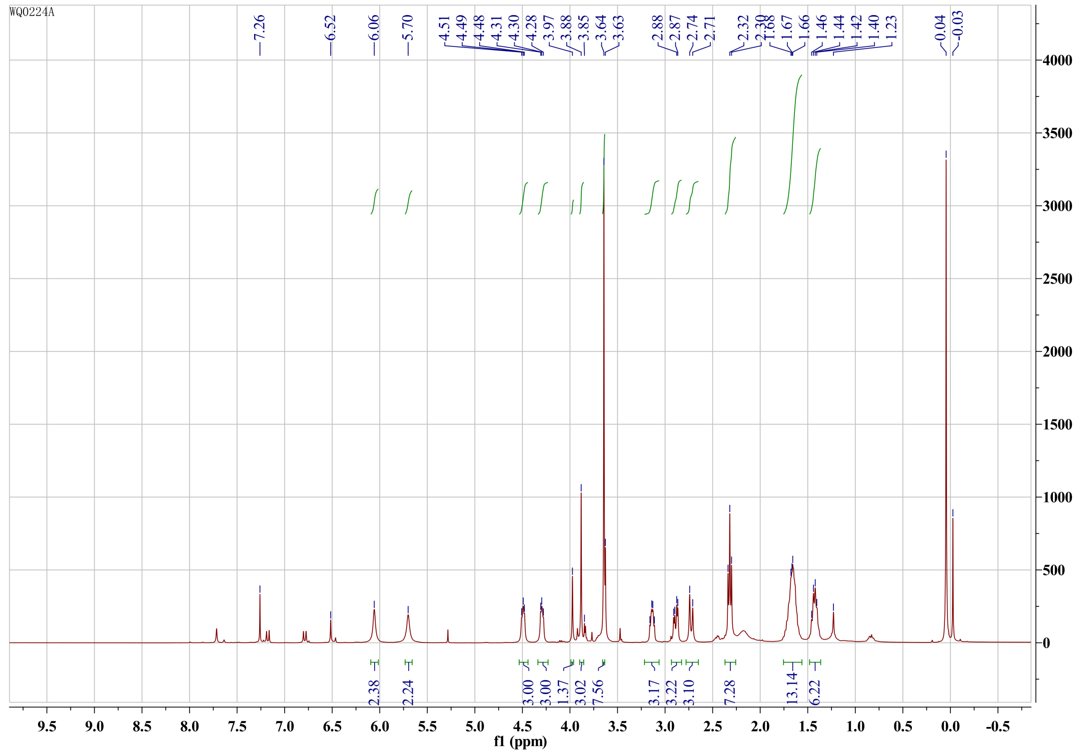

Figure S2. ^{1}H NMR spectrometer identification of Bio-Col.

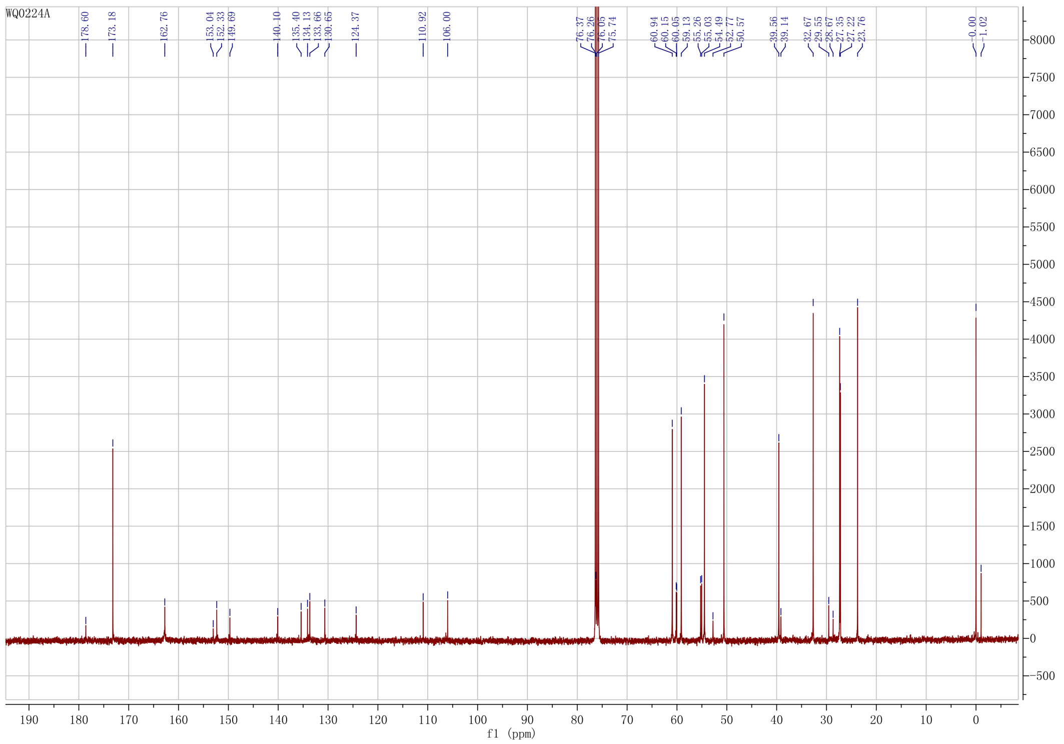

Figure S3. ^{13}C NMR spectrometer identification of Bio-Col.

Supplementary Tables

Table S1. Potential Targets for Colchicine by IFPTarget

ID	PDBID	Cvalue	TargetShortName	TargetFullName
1	3G58	16.195408	PDE4D_HUMAN	Human_phosphodiesterase_4d
2	1SLE	16.154512	SAV_STRAV	Streptavidin
3	1C8K	15.939321	PHS2_RABIT	Transferase
4	3ARW	15.786348	Q9AMP1_VIBHA	Chitinase_A
5	1DBJ	15.784373	FABF	Antibody_fab_fragment
6	4FLP	15.723901	BRDT_HUMAN	Bromodomain_testis-specific_protein(UNP_residues_21-137))
7	3B7J	15.711819	Q5G940_HELPY	HpFabZ
8	1Y2D	15.708879	PDE4D_HUMAN	Camp-specific_3',5'-cyclic_phosphodiesterase_4d
9	4GKI	15.667865	B0VD92_ACIBY	Aminoglycoside
10	4O2B	15.569482	TBA1B_BOVIN	Tubulin
11	3VWS	15.511154	Q6DLV0_9FLAV	Non-structural_protein_5
12	4N5G	15.477836	RXRA_HUMAN	Retinoic_acid_receptor_RXR-alpha_ligand_binding_domain
13	2A3B	15.468129	Q873X9_ASPFU	Chitinase
14	1QM4	15.455272	METL_RAT	Methionine_adenosyltransferase,_alpha_form
15	3O84	15.453923	B2HVG8_ACIBC	Peptide_arylation_enzyme_BasE_P45L
16	2WAJ	15.44479	MK10_HUMAN	Mitogen-Activated_Transferase_10,_HUMAN_JNK3
17	1KZ8	15.415054	F16P_PIG	Fructose-1,6-bisphosphatase
18	3R5N	15.3646	PPARG_HUMAN	Peroxisome_proliferator-activated_receptor_gamma
19	2HA7	15.280866	ACES_MOUSE	Acetylcholinesterase(S203A)
20	3AS0	15.243973	Q9AMP1_VIBHA	Chitinase_A(W275G_mutant)
21	1WBG	15.205281	THRB_HUMAN	Thrombin_light_chain
22	3OVN	15.181166	Q72498_9HIV1	POL_polyprotein
23	2AZ5	15.176651	TNFA_HUMAN	Tumor_necrosis_factor_(TNF-alpha)

24	4.00E+2 6	15.175396	BRAF_HUMAN	Serine/threonine-Transferase_B-raf(V600E)
25	4O72	15.172655	BRD4_HUMAN	Bromodomain-containing_protein_4_BRD4
26	3SZ1	15.165868	PPARG_HUMAN	Peroxisome_proliferator-activated_receptor_gamma
27	4AHU	15.131099	POL_HV1N5	INTEGRASE
28	3KWN	15.102108	CATS_HUMAN	Cys25Ser_mutant_of_cathepsin_S
29	3AS1	15.08595	Q9AMP1_VIBHA	Chitinase_A(W275G_mutant)
30	2G01	15.084189	MK08_HUMAN	Mitogen-activated_Transferase_8
31	1IUP	15.083796	P96965_PSEFL	Meta-cleavage_product_hydrolase
32	4ERK	15.068402	MK01_RAT	Extracellular_regulated_kinase_2
33	3NF8	15.050615	Q76353_9HIV1	Integrase(F185H,_C56S,_F139D_mutation)
34	4N9A	15.046138	DPO3B_ECOLI	E.coli_sliding_clamp,_DNA_polymerase_III _subunit_beta
35	3AS3	15.035046	Q9AMP1_VIBHA	Chitinase_A(W275G_mutant)
36	3OYN	15.031261	POL_FOAMV	Prototype_Foamy_Virus_(PFV)_intasome_N 224H_mutant
37	1W6Y	15.029419	SDIS_PSEPU	Steroid_delta-isomerase
38	4CFL	15.018072	Q6NXE4_HUMA N	N-terminal_bromodomain_of_human_BRD4
39	4LYS	15.013291	BRD4_HUMAN	Bromodomain-containing_protein_4_BRD4,
40	2XEZ	14.972245	CHK1_HUMAN	CHECKPOINT_KINASE_1_(CHK1)
41	3P5O	14.958964	BRD4_HUMAN	Bromodomain-containing_protein_4_BRD4(Human_Brd4)
42	1Y2F	14.939631	ZIPA_ECOLI	Cell_division_protein_zipa
43	2Q9M	14.920223	AMPC_ENTCL	AmpC_beta-Lactamase
44	2Y07	14.905611	ANMG	ANTI-NP_MURINE_GERMLINE
45	4MJQ	14.878182	DPO3B_ECOLI	E.coli_sliding_clamp,_DNA_polymerase_III _subunit_beta
46	3EBB	14.876539	PLAP_HUMAN	Phospholipase_A2-activating_protein
47	3CJ2	14.848783	POLG_HCVBK	HCV_NS5b_RNA_polymerase
48	3IEO	14.833867	CAH2_HUMAN	Carbonic_anhydrase_2

49	1L6M	14.825682	NGAL_HUMAN	Neutrophil_gelatinase-associated_lipocalin
50	3ARV	14.817199	Q9AMP1_VIBHA	Chitinase_A
51	1OBA	14.758552	LYCA_BPCP1	Lysozyme
52	4MSN	14.743291	PDE10_HUMAN	PDE10A2
53	1XN0	14.740553	PDE4B_HUMAN	Camp-specific_3',5'-cyclic_phosphodiesterase_4b
54	4FEA	14.715052	CASP7_HUMAN	Caspase-7
55	1Y2G	14.671824	ZIPA_ECOLI	Cell_division_protein_zipa
56	4P2T	14.660673	O36607_HHV8	KSHV_Protease
57	4E6C	14.660176	MK14_HUMAN	Mitogen-activated_Transferase_14
58	3DEI	14.617269	CASP3_HUMAN	Caspase-3
59	4LPH	14.611916	FPPS_HUMAN	Farnesyl_pyrophosphate_synthase,_human_FPPS
60	2QYL	14.608978	Q5T3Z8_HUMAN	PDE4B
61	3MXF	14.580472	BRD4_HUMAN	Bromodomain-containing_protein_4_BRD4(humand_BRD4(1))
62	1OYN	14.513029	PDE4D_HUMAN	Camp-specific_phosphodiesterase_pde4d2
63	3F8C	14.484841	A2RI36_LACLM	LmrR
64	4DSU	14.472032	RASK_HUMAN	GTPase_KRas,_isoform_2B
65	2XG5	14.46444	PAPD_ECOLX	E.COLI_P_PILUS_CHAPERONE-SUBUNIT_COMPLEX_PAPD-PAPH
66	4NBL	14.438158	CASP6_HUMAN	ProCaspase-6
67	1E1Y	14.422567	PHS2_RABIT	Transferase
68	4UYH	14.401581	BRD2_HUMAN	BRD2_N-terminal_bromodomain
69	2QYN	14.357333	PDE4D_HUMAN	PDE4D
70	4EH2	14.345509	MK14_HUMAN	Mitogen-activated_Transferase_14
71	1XMU	14.276976	PDE4B_HUMAN	Camp-specific_3',5'-cyclic_phosphodiesterase_4b
72	2K0X	14.267177	ACPX_STRCO	Actinorhodin_holo_acyl_carrier_protein
73	4ABV	14.226688	TTHY_HUMAN	TRANSTHYRETIN

74	1XLZ	14.206004	PDE4B_HUMAN	Camp-specific_3',5'-cyclic_phosphodiesterase_4b
75	3ARZ	14.195946	Q9AMP1_VIBHA	Chitinase_A
76	3M1J	14.194715	CAH2_HUMAN	Carbonic_anhydrase_2
77	1XOQ	14.064639	PDE4D_HUMAN	Camp-specific_3',5'-cyclic
78	1W80	14.056063	APCA2	Adapter-related_protein_complex_2_alpha_2
79	1B42	14.025743	PAP2_VACCV	Vp39
80	4NBK	14.007546	CASP6_HUMAN	ProCaspase-6
81	1N5R	13.914145	ACES_MOUSE	Acetylcholinesterase
82	4FZJ	13.843853	PANC_MYCTU	Pantothenate_synthetase_(T2A,_E77G_mutation)
83	3ARY	13.841335	Q9AMP1_VIBHA	Chitinase_A
84	4HXR	13.731911	BRD4_HUMAN	Bromodomain-containing_protein_4_BRD4
85	3WKB	13.667207	HYES_HUMAN	Soluble_epoxide_hydrolase_2
86	4WCU	13.653337	PDE4D_HUMAN	CAMP-specific_3',5'-cyclic_phosphodiesterase_4D_PDE4D
87	4KZB	13.64751	AMPC_ECOLI	AmpC_beta-Lactamase
88	3BGQ	13.637585	PIM1_HUMAN	PIM-1
89	3K27	13.59612	EED_HUMAN	Polycomb_protein_EED
90	3ARX	13.59234	Q9AMP1_VIBHA	Chitinase_A
91	4DE1	13.551861	Q9L5C8_ECOLX	Beta-lactamase
92	2HA4	13.534146	ACES_MOUSE	Acetylcholinesterase(S203A)
93	2QH6	13.49937	ESR1_HUMAN	Estrogen_receptor
94	2IUZ	13.465527	Q873X9_ASPFU	Chitinase
95	4A9M	13.449446	BRD2_HUMAN	N-TERMINAL_BROMODOMAIN_OF_HUMAN_BRD2
96	1BN4	13.417478	CAH2_HUMAN	Carbonic_anhydrase_2
97	3NAM	13.398555	B6CAM1_RABIT	SERCA1a,_SR_Ca(2+)-ATPase
98	3G3N	13.346646	PDE7A_HUMAN	PDE7A_catalytic_domain
99	4O74	13.316369	BRD4_HUMAN	Bromodomain-containing_protein_4_BRD4

100	3ZST	13.294551	PEP1A_STRCO	PUTATIVE_GLUCANOHYDROLASE_PEP1A_GLGE_ISOFORM_1
101	1RV1	13.279514	MDM2_HUMAN	Ubiquitin-protein_ligase_e3_mdm2
102	4N6G	13.271523	CASP6_HUMAN	ProCaspase-6
103	4HBX	13.232351	BRD4_HUMAN	Bromodomain-containing_protein_4_BRD4
104	1T4J	13.231334	PTN1_HUMAN	Protein-tyrosine_phosphatase,_non-receptor_type
105	4N6Y	13.217312	PIM1_HUMAN	Serine/threonine-Transferase_pim-1
106	3GXL	13.215693	TGFR1_HUMAN	TGF-beta_receptor_type-1,_ALK-5_kinase
107	3U5L	13.209298	BRD4_HUMAN	Bromodomain-containing_protein_4_BRD4
108	4FCD	13.204321	PDE10_HUMAN	CAMP_and_cAMP-inhibited_cGMP_3',5'-cyclic_phosphodiesterase_10A
109	4J74	13.200628	MDM2_XENLA	E3_ubiquitin-protein_ligase_Mdm2_(I50L,_P92H,_L95I_mutation)
110	3VBY	13.180168	PIM1_HUMAN	Serine/threonine-Transferase_pim-1
111	4N5D	13.164074	CASP6_HUMAN	Caspase-6
112	4QZS	13.129154	BRD4_HUMAN	Bromodomain-containing_protein_4_BRD4
113	3HQW	13.126483	PDE10_RAT	CAMP_and_cAMP-inhibited_cGMP_3,5-cyclic_phosphodiesterase_10A
114	3G4K	13.101542	PDE4D_HUMAN	Human_phosphodiesterase_4d
115	2FU8	13.024117	BLA1_XANMA	L1_MBL
116	1P5E	13.016047	CDK2_HUMAN	CDK2_cyclin_dependent_kinase_2
117	3ZYU	12.98847	BRD4_HUMAN	HUMAN
118	4A9I	12.985852	BRD2_HUMAN	N-TERMINAL_BROMODOMAIN_OF_HUMAN_BRD2
119	4MEN	12.981179	BRD4_HUMAN	Bromodomain-containing_protein_4_BRD4
120	4CTK	12.975599	A9LIE0_9FLAV	Dengue_virus_3_non-structural_protein_5
121	1Y2J	12.969619	PDE4B_HUMAN	Camp-specific_3',5'-cyclic_phosphodiesterase_4b
122	4NTJ	12.951948	P2Y12_HUMAN	P2Y12R

123	3F6H	12.90868	Q8F3Q1_LEPIN	Alpha-isopropylmalate_synthase
124	4MRA	12.898852	PYGM_RABIT	Transferase,_muscle_form
125	2LHA	12.88904	SYT1_HUMAN	Synaptotagmin-1
126	5CFB	12.888369	GLRA3_HUMAN	Human Glycine Receptor alpha-3
127	4A4G	12.847745	SMN_HUMAN	Tudor_domain_of_SURVIVAL_MOTOR_NEURON_PROTEIN
128	3U5J	12.821589	BRD4_HUMAN	Bromodomain-containing_protein_4_BRD4
129	4HGE	12.819681	JAK2_HUMAN	Tyrosine-Transferase_JAK2
130	4NH8	12.808615	HS90A_HUMAN	Heat_shock_protein_HSP90-alpha
131	4B80	12.784443	ACES_MOUSE	Acetylcholinesterase
132	1HTY	12.77574	MAN2_DROME	Alpha-mannosidase_ii
133	3EBP	12.76965	PYGM_RABIT	Transferase,_muscle_form
134	3WKD	12.768015	HYES_HUMAN	Soluble_epoxide_hydrolase_2
135	3PWD	12.761925	CSK2A_MAIZE	Casein_kinase_II_subunit_alpha;_CK2
136	4N99	12.759496	DPO3B_ECOLI	E.coli_sliding_clamp,_DNA_polymerase_III_subunit_beta
137	2XF0	12.758574	CHK1_HUMAN	CHECKPOINT_KINASE_1_(CHK1)
138	3O57	12.739399	PDE4B_HUMAN	CAMP-specific_3',5'-cyclic_phosphodiesterase_4B,_PDE4B
139	3TF6	12.735669	NGAL_HUMAN	Mammalian_antibacterial_protein
140	3QSB	12.722759	DPO3B_ECOLI	DNA_polymerase_III_subunit_beta

Table S2. Ligand-based target prediction using SEA (Keiser et al., 2009).

ID	Potent Targets	Score
1	Tubulin beta-8 chain	1.05E-62
2	Tubulin beta-3 chain	1.05E-62
3	Tubulin beta-4 chain	1.05E-62
4	Tubulin beta-2 chain	1.05E-62
5	Tubulin alpha-3 chain	1.98E-55
6	Tubulin beta-1 chain	1.98E-55
7	Tubulin gamma-1 chain	1.98E-55
8	Tubulin alpha-1 chain	2.67E-39
9	Tubulin alpha chain	1.02E-30
10	Tubulin beta-5 chain	3.43E-28
11	Tubulin beta chain	1.77E-26
12	Glycine receptor alpha-3 chain	2.57E-52
13	Glycine receptor alpha-2 chain	2.30E-49
14	Glycine receptor alpha-1 chain	6.44E-20
15	Melatonin receptor 1B	1.20E-07
16	Melatonin receptor 1A	7.83E-07
17	Steryl-sulfatase precursor	3.74E-02
18	Carbonic anhydrase XII	1.08E-01
19	Quinone reductase 2	1.13E+00
